# Dietary supplementation with *Lactiplantibacillus plantarum* P-8 improves the growth performance and gut microbiota of weaned piglets

**DOI:** 10.1128/spectrum.02345-22

**Published:** 2024-01-03

**Authors:** Jie Yu, Bin Zuo, Qi Li, Feiyan Zhao, Junjun Wang, Weiqiang Huang, Zhihong Sun, Yongfu Chen

**Affiliations:** 1 Key Laboratory of Dairy Biotechnology and Engineering, Ministry of Education, Inner Mongolia Agricultural University, Huhhot, China; 2 Key Laboratory of Dairy Products Processing, Ministry of Agriculture and Rural Affairs, Inner Mongolia Agricultural University, Huhhot, China; 3 State Key Laboratory of Animal Nutrition, College of Animal Science and Technology, China Agricultural University, Beijing, China; Iowa State University, Ames, Iowa, USA

**Keywords:** weaned piglets, microbiota, *Lactiplantibacillus plantarum*, growth performance, antibiotic resistance genes

## Abstract

Weaning is a stressful event in the pig life cycle. We hypothesized that probiotics could be potential alternatives to antibiotics for promoting growth and ameliorating stress in weaning piglets via gut microbiota modulation and, thus, investigated the beneficial effects of dietary probiotic supplementation in weaning pigs. Ninety weaning piglets (Landrace × large white, 45 males and 45 females, 25 days of age) were randomized into three dietary treatments (30 piglets/treatment, divided into five replicates/treatment, i.e., six piglets/replicate) in this 28-day trial: control (C group, basal diet); probiotic [lactic acid bacteria (LAB) group, basal diet plus *Lactiplantibacillus plantarum* P-8]; and antibiotic (A group; basal diet plus chlortetracycline). The piglets’ growth performance [average daily gain, average daily feed intake (ADFI), and feed conversion ratio (FCR)], immune and antioxidant markers, ileal mucosal morphology, and ileal and colonic microbiomes were compared among treatment groups. Compared to the C and A groups, probiotic supplementation significantly decreased the ADFI, FCR, and ileal mucosal crypt depth while increasing the villus height-to-crypt depth ratio, hepatic glutathione peroxidase and catalase activities, and serum levels of interleukin-2. Both probiotic and antibiotic treatments modulated the piglets’ gut microbiomes, with more *L. plantarum* in the LAB group and more *Eubacterium rectale* and *Limosilactobacillus reuteri* in the A group. Probiotic supplementation significantly increased the relative abundance of genes encoding the acetylene, galactose, and stachyose degradation pathways, potentially enhancing nutrient absorption, energy acquisition, and growth performance. Probiotics are effective alternatives to antibiotics for promoting the health of piglets, possibly via gut microbiome modulation.

**IMPORTANCE:**

Weaning impacts piglet health, performance, and mortality. Antibiotic treatment during weaning can mitigate the negative effects on growth. However, antibiotic use in livestock production contributes to the emergence of antibiotic resistance, which is a threat to global public health. This comprehensive study describes the gut microbial composition and growth performance of weaned piglets after dietary supplementation with *Lactiplantibacillus plantarum* P-8 or antibiotics. *L. plantarum* P-8 ameliorated stress and improved antioxidant capacity and growth performance in weaned piglets, accompanied by gut microbiota improvement. *L. plantarum* P-8 is an effective substitute for antibiotics to promote the health of weaned piglets while avoiding the global concern of drug resistance.

## INTRODUCTION

Pigs are one of the most important economic livestock animals worldwide. Weaning is a critical event in the pig life cycle; it impacts piglet health, performance, and mortality (1). At weaning, piglets are removed from their mothers and experience nutritional, physiological, and psychological stressors. Such stressors induce post-weaning diarrhea, transient anorexia, intestinal inflammation, and imbalanced gut microbiota (2, 3). Treatment with antibiotics during weaning can mitigate the negative effects on growth. Observations of significant changes in the gut microbiota of piglets in response to in-feed antibiotics and the subsequent shift in their fecal bacterial microbiota composition and function suggest that antibiotics enhance the growth of and alleviate weaning stress in livestock animals via gut microbiota regulation (4, 5). However, antibiotic use in livestock production contributes to the emergence of antibiotic resistance, which is a threat to global public health (6, 7). There is an urgent need to seek alternative non-antibiotic strategies to maintain gut homeostasis and combat gastrointestinal infections associated with weaning in piglets. Alternative treatments that have been evaluated for their beneficial effects in restoring the gastrointestinal balance and reducing weaning stress include zinc oxide (8), organic acids (9), essential oils (10), N-acetyl-D-glucosamine (11), and prebiotics and probiotics (12).

The gut microbiome plays crucial roles in a variety of important physiological processes in host metabolism and health, such as gut maturation, nutrient absorption, immune modulation, vitamin synthesis, and pathogen resistance (13, 14). It is a complex and dynamic ecosystem, the composition of which changes continually in response to new microbes that enter the gut environment. Factors such as lifestyle, diet, and antibiotic use influence the gut microbial community. Gut dysbiosis is associated with the onset and pathogenesis of many inflammatory diseases and infections (15). Research on the gut microbiome is increasing rapidly due to growing interest in the function of the gut microbiota in human and animal health.

In recent years, probiotics have emerged as one of the most effective alternatives to antibiotics for enhancing animal health and growth performance through modulating the gut microbiota; their effects on weaned piglets have been widely documented. For example, probiotics play an important role in the formation or establishment of well-balanced, indigenous intestinal microbiota in piglets. For instance, dietary supplementation with *Enterococcus faecalis* UC-100 increases body weight (BW) gain, decreases the feed-to-gain ratio, and reduces incidences of diarrhea in weaned pigs compared to those fed only a basal diet. Furthermore, piglets that receive an *E. faecalis* supplement have reduced abundances of Fibrobacterota and 12 genera, including *Fibrobacter* and some opportunistic pathogens, compared to those fed only a basal diet (16). Other studies have reported that dietary supplementation with *Lacticaseibacillus rhamnosus* GG alleviates diarrhea in weaned piglets by improving the intestinal microbiota (17) and promotes lymphocyte proliferation and Th1 differentiation in early weaned piglets (18). Wang et al. (19) reported that *Lactiplantibacillus plantarum* PFM105 improves the growth performance of weaned piglets, potentially via modulating the piglets’ gut microbiota and increasing the abundances of symbiotic and beneficial bacteria. Collectively, these findings support probiotic lactic acid bacteria (LAB) as potential alternatives to antibiotics in pig farming to promote animal health and improve growth performance.

The *L. plantarum* P-8 strain is a probiotic bacterium isolated from naturally fermented yogurt in the Inner Mongolia Autonomous Region of China (20). Based on the desirable properties conferred by *L. plantarum* P-8, including high tolerance to acid and bile, good aggregation activity, and antibacterial activity, it was selected for further *in vivo* experiments (21). A large number of human and animal trials have shown beneficial effects of *L. plantarum* P-8 supplementation, including the inhibition of pathogenic bacteria; enhancement of secretory immunoglobulin (Ig) A levels and antioxidant capacity in human adults (22); promotion of weight gain, feed intake, and IgA and IgG levels in broiler chickens (23); and alleviation of canine diarrhea via regulating the gut microbiota (24).

In the present study, we investigated the health-promoting effects of *L. plantarum* P-8 supplementation on the growth performance, immune markers, and antioxidant capacity of weaned piglets. To elucidate the probiotic mechanism, we also investigated potential links between the ileal and colonic microbiomes and various health parameters. Finally, antibiotic and probiotic intervention-induced changes in the gut microbiome antibiotic resistance gene (ARG) profile were compared.

## RESULTS

### Piglet growth performance and ileal morphology

In total, 90 piglets were weaned at 25 days old (Landrace × large white, 7.75 ± 0.08 kg) and were randomly allocated to three groups for the 28-day trial: control (C group, basal diet); probiotic (LAB group, basal diet plus *L. plantarum* P-8); and antibiotic (A group, basal diet plus chlortetracycline). During the first 2 weeks, no significant differences were observed in the BW, average daily gain (ADG), average daily feed intake (ADFI), and feed conversion ratio (FCR, feed consumed/weight gain) across the groups, except that the ADG was significantly higher in the LAB group than in the C and A groups (Table 1, *P* < 0.05). During the latter 2 weeks, the ADFI and FCR were significantly reduced in piglets in the LAB group compared to the C and A groups (Table 1, *P* < 0.05). Moreover, the overall BW at day 28 and ADG over the 4-week intervention of the LAB group were significantly (*P* < 0.05) and non-significantly (*P* > 0.05) greater, respectively, than those of the C and A groups. These results suggest that probiotic supplementation enhanced the piglets’ growth performance and that less feed was required for the piglets in the LAB group to achieve the same growth rate as in the other two groups.

**TABLE 1:** Effects of probiotics and antibiotics on growth performance of weaning piglets^*a*^

Group	C	A	LAB	*P* value
BW (kg)^*d*^				
Day 0	7.75 ± 1.00	7.75 ± 1.01	7.75 ± 1.01	0.98
Day 14	11.58 ± 1.36	11.71 ± 0.97	11.97 ± 1.37	0.81
Day 28	17.65 ± 2.01	18.09 ± 1.60	18.52 ± 2.33^*b*^	0.82
ADG (kg/day)^*d*^				
Days 0–14	273.54 ± 29.27	282.45 ± 31.52	300.42 ± 35.99^*b*^	0.33
Days 14–28	433.79 ± 67.72	455.33 ± 51.62	468.33 ± 75.67	0.44
Days 0–28	353.66 ± 44.45	368.89 ± 31.17	384.37 ± 54.90^*b*^	0.54
ADFI (kg/day)^*d*^				
Days 0–14	505.55 ± 59.46	490.57 ± 49.83	495.00 ± 63.93	0.85
Days 14–28	843.87 ± 73.03	829.32 ± 115.31	735.20 ± 101.11^*b*^^,^^*c*^	<0.05
Days 0–28	686.82 ± 60.41	659.95 ± 76.06	606.95 ± 59.01^*b*^	<0.05
FCR^*d*^				
Days 0–14	1.82 ± 0.08	1.76 ± 0.15	1.71 ± 0.09	0.075
Days 14–28	1.95 ± 0.22	1.85 ± 0.11	1.64 ± 0.19^*b*^^,^^*c*^	<0.01
Days 0–28	1.90 ± 0.15	1.81 ± 0.09	1.67 ± 0.14^*b*^^,^^*c*^	<0.01

^*a*^ Groups: C, negative control without receiving antibiotics or probiotics; A, antibiotic (chlortetracycline) given orally; LAB, probiotic lactic acid bacteria (*Lactiplantibacillus plantarum* P-8) given orally. Data are expressed as mean ± standard deviation.

^*b*^ Those parameters were compared between groups using Wilcoxon tests. Compared with control, *P < 0.05.

^*c*^ Those parameters were compared between groups using Wilcoxon tests. Compared with antibiotics, #*P* < 0.05.

^*d*^ Abbreviations: ADFI, average daily feed intake; ADG, average daily gain; BW, body weight; FCR, feed conversion ratio.

**Fig 1:**
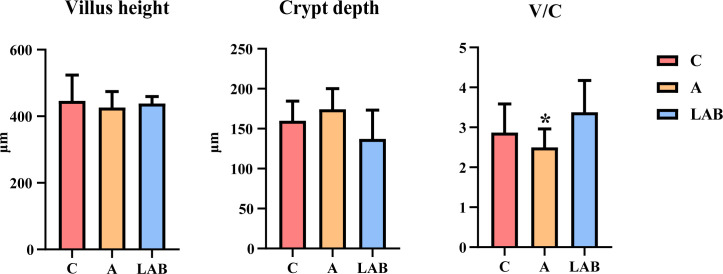
Effects of probiotics and antibiotics on ileal villus height (μm) and crypt depth (μm). Groups: C, negative control not given antibiotics or probiotics; A, antibiotic (chlortetracycline) given orally; LAB, probiotic lactic acid bacteria (*Lactiplantibacillus plantarum* P-8) given orally. Error bars represent the SD. *P < 0.05. *V*:*C*, villus height-to-crypt depth.

No significant differences were found in the ileal villus height and crypt depth across the three groups (*P* > 0.05, Fig. 1); however, the LAB group showed a non-significant decrease in crypt depth compared to the C and A groups. Moreover, the ileal villus height-to-crypt depth (*V*:*C*) ratio was significantly reduced in the A group compared to the C and LAB groups (*P* < 0.05). Meanwhile, the *V*:*C* ratio was distinctly, albeit non-significantly, higher in the LAB group than in the C group (*P* > 0.05).

### Antioxidant and immune factor levels

To investigate the roles of probiotic supplementation in protecting against weaning-associated oxidative stress, the serum and hepatic total antioxidant capacities (T-AOCs) and catalase (CAT), superoxide dismutase (SOD), glutathione peroxidase (GSH-Px), and malondialdehyde (MDA) levels were measured at day 28 (Table 2). Liver CAT activity was significantly lower in the A and LAB groups than in the C group (*P* < 0.05). Liver SOD and GSH-Px activities were significantly lower in the A group than in the C and LAB groups (*P* < 0.05). No significant differences were observed in the liver T-AOC and MDA levels among the groups (*P* > 0.05). Moreover, no significant differences were observed in the serum levels of most measured antioxidant indices (T-AOC, CAT, SOD, and MDA; *P* > 0.05), except that the A group had the lowest serum GSH-Px activity among the three groups (A group: 1,208.48 ± 246.15 U/mL; C group: 1,250.25 ± 270.99 U/mL; LAB group: 1,221.06 ± 282.1 U/mL; Table 2).

**TABLE 2:** Effects of *Lactiplantibacillus plantarum* P-8 intake on hepatic and serum antioxidant indices of piglets^*a*^

Group^*c*^	C	A	LAB	*P* value
Hepatic antioxidant indices				
T-AOC (U/mL)	1.48 ± 0.48	1.36 ± 0.42	2.10 ± 0.94	0.26
CAT (U/mL)	10.03 ± 2.88	5.96 ± 1.45^*b*^	5.97 ± 2.58^*b*^	<0.01
SOD (U/mL)	9.09 ± 1.79	7.76 ± 1.96^*b*^	11.63 ± 3.35	<0.05
GSH-Px (U/mL)	66.67 ± 29.86	36.37 ± 8.03^*b*^	69.50 ± 31.64	<0.05
MDA (nmol/mL)	0.35 ± 0.11	0.29 ± 0.07	0.32 ± 0.08	0.51
Serum antioxidant indices				
T-AOC (U/mL)	8.62 ± 3.54	7.22 ± 3.01	10.26 ± 4.94	0.36
CAT (U/mL)	59.66 ± 3.058	64.61 ± 23.49	58.62 ± 32.83	0.74
SOD (U/mL)	79.06 ± 30.55	76.94 ± 20.04	68.92 ± 23.70	0.70
GSH-Px (U/mL)	1,250.25 ± 270.99	1,208.48 ± 246.15^*b*^	1,221.06 ± 282.1	<0.05
MDA (nmol/mL)	4.84 ± 0.51	5.35 ± 1.80	4.57 ± 1.38	0.44
Serum immune indices				
IgA (mg/mL)	1.12 ± 0.08	1.32 ± 0.13^*b*^	1.14 ± 0.12	<0.01
IgG (mg/mL)	20.24 ± 0.82	21.94 ± 0.80^*b*^	20.38 ± 1.23	<0.01
IgM (mg/mL)	2.37 ± 0.07	2.51 ± 0.06^*b*^	2.38 ± 0.11	<0.01
IFN-γ (pg/mL)	49.95 ± 13.69	47.95 ± 13.82	54.38 ± 11.10	0.47
TNF-α (pg/mL)	60.30 ± 8.22	60.42 ± 18.91	65.71 ± 19.80	0.70
IL-1β (pg/mL)	30.06 ± 7.24	30.98 ± 10.69	32.93 ± 7.82	0.49
IL-2 (pg/mL)	37.26 ± 6.18	40.02 ± 19.37	39.24 ± 17.29	0.82
IL-4 (pg/mL)	12.97 ± 3.45	10.09 ± 3.77	9.16 ± 2.91^*b*^	0.06
IL-6 (pg/mL)	114.94 ± 12.97	119.10 ± 35.60	118.21 ± 32.54	0.82
IL-10 (pg/mL)	31.42 ± 5.72	27.72 ± 4.83	26.56 ± 5.30	0.07

^*a*^ Groups: C, negative control without receiving antibiotics or probiotics; A, antibiotic (chlortetracycline) given orally; LAB, probiotic lactic acid bacteria (*Lactiplantibacillus plantarum* P-8) given orally. Data are expressed as mean ± SD.

^*b*^ Those parameters were compared between groups using Wilcoxon tests. Compared with control, *P < 0.05.

^*c*^ Abbreviations: CAT, catalase; GSH-Px, glutathione peroxidase; IFN-γ, interferon-alpha; Ig, immunoglobulin; IL, interleukin; MDA, malondialdehyde; SOD, superoxide dismutase; T-AOC, total antioxidant capacity; TNF-α, tumor necrosis factor-alpha.

Next, the effects of antibiotic and probiotic treatments on host immunity were evaluated by measuring the serum levels of IgG, IgA, IgM, tumor necrosis factor-alpha (TNF-α), interleukin (IL)-2, IL-4, IL-6, IL-10, IL-1β, and interferon-gamma (IFN-γ) (Table 2). IgA, IgG, and IgM levels significantly differed among the groups (*P* < 0.01); in particular, their levels were significantly greater in the A group than in the C group (*P* < 0.05). The LAB group had the lowest IL-4 level among the three groups, which was significantly lower than that of the C group (*P* < 0.05). No significant differences were observed in the IFN-γ, TNF-α, IL-1β, IL-2, IL-6, or IL-10 levels across the three groups (*P* > 0.05).

### Bacterial community richness and biodiversity

#### Probiotic-mediated modulation of gut microbial diversity and structure

**Fig 2:**
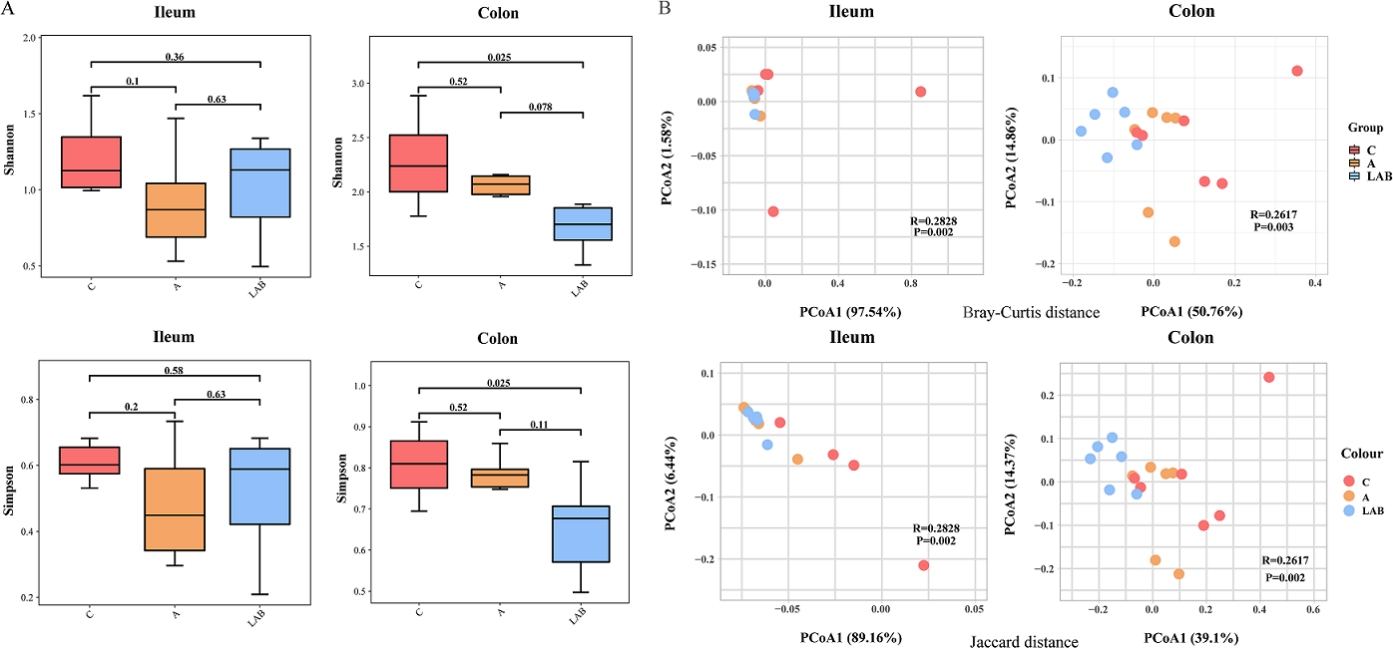
Alpha and beta diversity of the ileal and colonic microbiota of weaning piglets. Groups: C, negative control not given antibiotics or probiotics; A, antibiotics (chlortetracycline) given orally; LAB, probiotic lactic acid bacteria (*Lactiplantibacillus plantarum* P-8) given orally. (**A**) The Simpson and Shannon index values of the colonic and ileal microbiota. All values are expressed as mean ± SD. (**B**) Principal coordinate analysis (Bray–Curtis and Jaccard distances) score plot of the colonic and ileal microbiota. *R* and *P* values generated by analysis of similarities are shown. PCoA, principal coordinate analysis.

After quality control and removal of chimeric reads, 39,402,874 high-quality sequences were obtained across all ileal and colonic microbiota samples. The Shannon and Simpson indices of the ileal and colonic microbiota of weaned piglets were compared among the three groups (Fig. 2A). Both indices were significantly lower in the colonic microbiota of the LAB group than in the C group (all *P* = 0.025), whereas no significant differences were observed in the ileal microbiota across the three groups (*P* > 0.05).

To evaluate the effects of probiotic and antibiotic interventions on the beta diversities of the ileal and colonic microbiota in weaned piglets, principal coordinate analyses (PCoAs; Bray–Curtis and Jaccard distances) were performed based on the taxonomic distributions and abundances of the respective microbiota. Calculations based on the Bray–Curtis distance and Jaccard distance achieved similar results. On the PCoA score plots, symbols representing the ileal and colonic microbiota of probiotic- and antibiotic-fed piglets clustered closer together than those representing samples from the C group, suggesting that the ileal and colonic bacterial microbiota of the LAB and A groups were more similar to each other than to those of the C group (Fig. 2B). However, a greater difference was observed between the beta diversities (Bray–Curtis distance and Jaccard distance) of the colonic microbiota than the ileal microbiota of the LAB and A groups.

The results of analysis of similarities (ANOSIM) confirmed the significant differences in the structure and composition of the ileal and colonic microbiota across the three groups. However, the *R* values were relatively small, possibly due to the small sample size. The results of the microbiota diversity analyses suggested that gut microbiota modulation was attributable to probiotic and antibiotic administrations.

#### Differences in piglet gut microbiota composition among treatment groups

**Fig 3:**
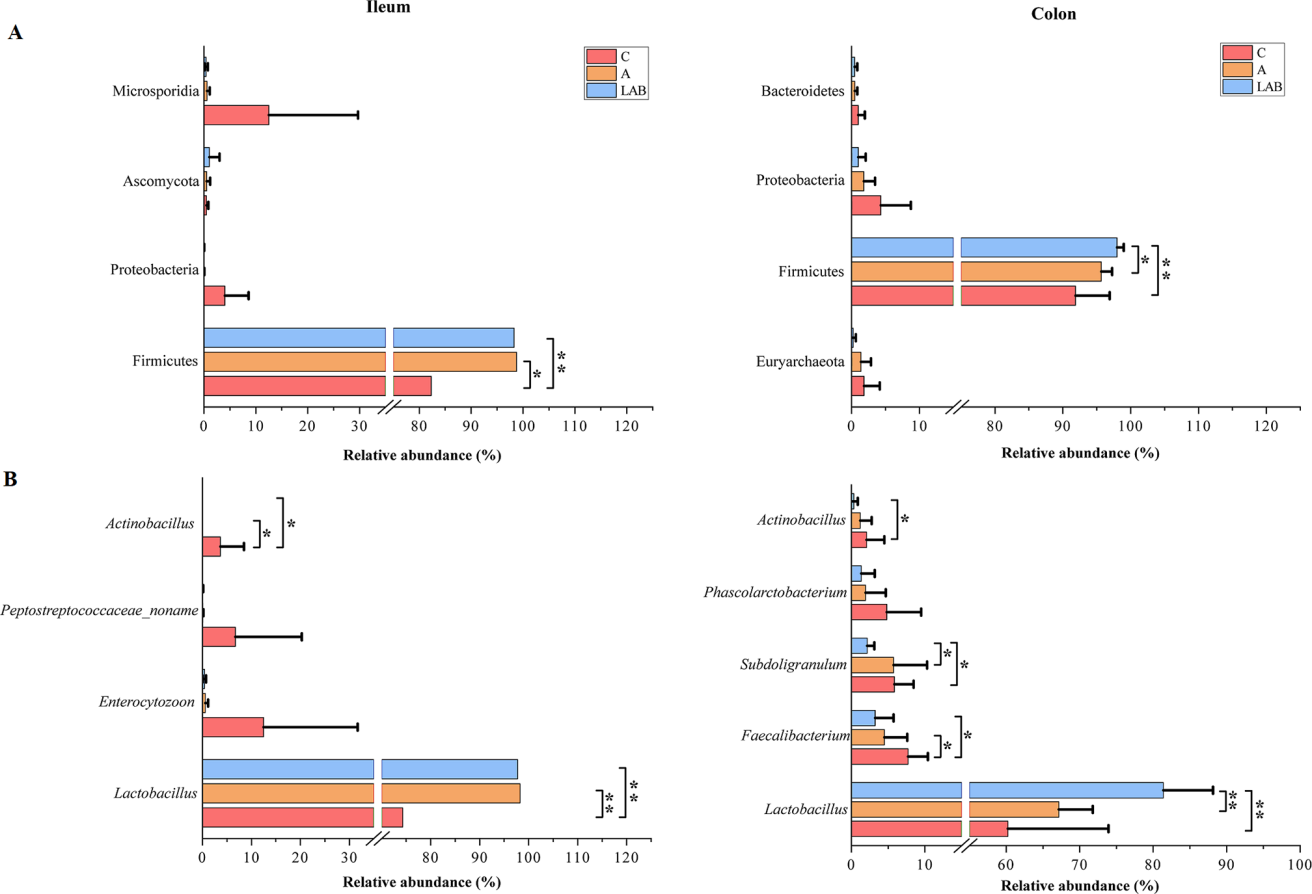
Differential composition of ileal and colonic microbiota in three groups of weaning piglets at the (**A**) phylum and (**B**) genus levels. Groups: C, negative control not given antibiotics or probiotics; A, antibiotic (chlortetracycline) given orally; LAB, probiotic lactic acid bacteria (*Lactiplantibacillus plantarum* P-8) given orally. All values are expressed as mean ± SD. *P < 0.05, **P < 0.01.

The relative abundances of bacterial genomes were calculated based on the average coverage per metagenomics bin normalized to the number of total reads in each sample. At the phylum level (Fig. 3A), Firmicutes was predominant in both the colon (93.10%) and ileum (95.16%). The relative abundance of Firmicutes was significantly lower in the C group than in the LAB group in both the ileum and colon (all *P* < 0.01); however, in the A group, the abundance was significantly lower in the colon (*P* < 0.05) but not the ileum (*P* > 0.05). The minor phyla differed between the ileal (Proteobacteria, Microsporidia, and Ascomycota) and colonic (Proteobacteria, Bacteroidetes, and Euryarchaeota) microbiota (Fig. 3A).

Next, the genus-level differences in the ileal and colonic bacterial microbiota compositions among the treatment groups were explored (Fig. 3B). The predominant genus was *Lactobacillus* in both the ileal (80.00%) and colonic (67.16%) microbiota. By contrast, the abundances of other genera (*Actinobacillus*, *Subdoligranulum*, and *Faecalibacterium*) were significantly lower in both the colonic and ileal microbiota in all three groups. In the ileum, the *Lactobacillus* abundance was significantly greater in the LAB and A groups than in the C group (all *P* < 0.01). The opposite trend was observed for *Actinobacillus* (*P* < 0.05). In the colon, *Lactobacillus* was significantly more abundant in the LAB group than in the A and C groups (*P* < 0.01); no differences were observed between the A and C groups. Meanwhile, the abundances of *Actinobacillus*, *Subdoligranulum*, and *Faecalibacterium* were significantly lower in the LAB group than in the C group (all *P* < 0.05). Finally, antibiotic supplementation significantly lowered the relative abundance of *Faecalibacterium* (*P* < 0.05).

### Identification of biomarkers in the treatment groups

**Fig 4:**
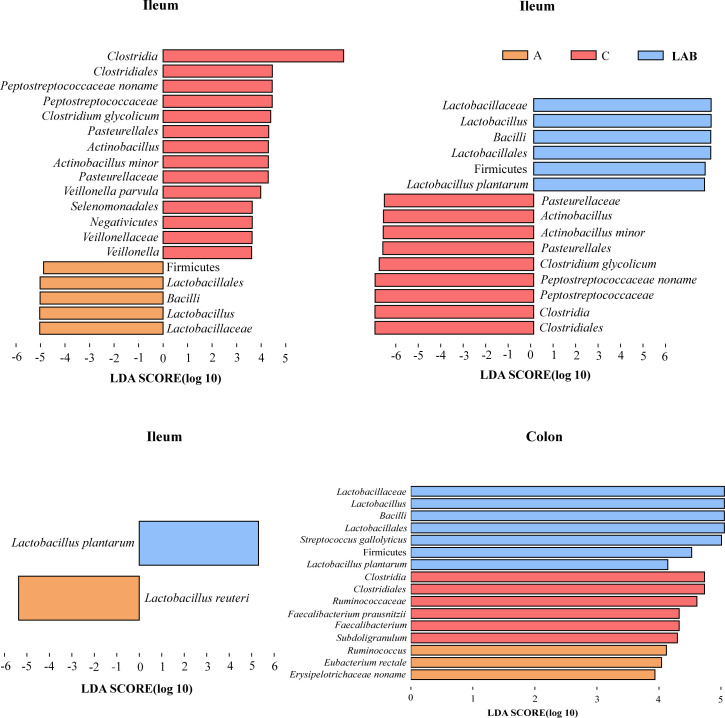
Linear discriminant analysis (LDA) effect size analysis of colonic and ileal microbial communities in weaning piglets. Groups: C, negative control not provided antibiotics or probiotics; A, antibiotics (chlortetracycline) given orally; LAB, probiotic lactic acid bacteria (*Lactiplantibacillus plantarum* P-8) given orally. The length of the horizontal bar represents the LDA score. The cut-off LDA score was 3, indicating a significant difference in species composition between groups.

Differentially abundant taxa among the LAB, C, and A groups were identified based on linear discriminant analysis effect size (LEfSe). LEfSe identified a number of differential biomarkers in the ileal microbiota (Fig. 4). At the species level, *Actinobacillus minor*, *Clostridium glycolicum*, and *Veillonella parvula* were enriched in the C group; *L. plantarum* was enriched in the LAB group but low in the A group (Fig. 4A and B); and *Limosilactobacillus reuteri* was enriched in the A group (Fig. 4C). In the colonic microbiota, 16 significantly differential features were identified across the three treatment groups (Fig. 4D). At the species level, *Streptococcus gallolyticus* and *L. plantarum* were enriched in the LAB group; *Faecalibacterium prausnitzii* was enriched in the C group; and *Eubacterium rectale* was enriched in the A group (Fig. 4D).

### Comparison of predicted gut microbiota function among the treatment groups

**Fig 5:**
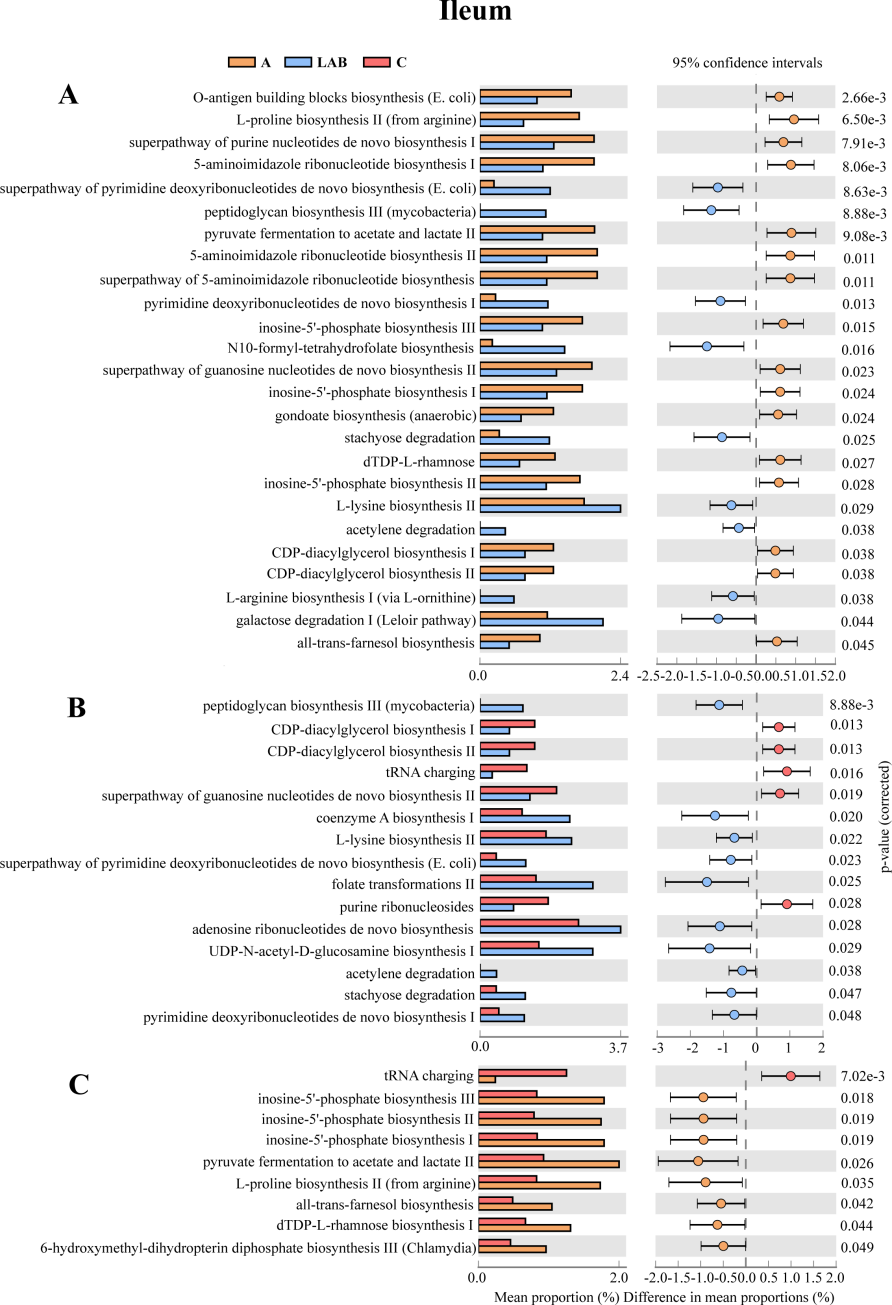
Differentially abundant metabolic pathways encoded in the ileal microbial functional metagenomes. Groups: C, negative control not provided antibiotics or probiotics; A, antibiotics (chlortetracycline) given orally; LAB, probiotic lactic acid bacteria (*Lactiplantibacillus plantarum* P-8) given orally. Pairwise comparisons between the (**A**) A and LAB groups, (**B**) LAB and C groups, and (**C**) A and C groups.

The Kyoto Encyclopedia of Genes and Genomes (KEGG) annotated functional metagenomes of the three treatment groups were compared. For the ileal microbiota (Fig. 5), the LAB group had significantly higher relative abundances of genes encoding pathways related to peptidoglycan biosynthesis III, superpathway of pyrimidine deoxyribonucleotide *de novo* biosynthesis, pyrimidine deoxyribonucleotide *de novo* biosynthesis I, coenzyme A biosynthesis I, L-lysine biosynthesis II, L-arginine biosynthesis II, UDP-N-acetyl-D-glucosamine biosynthesis I, adenosine ribonucleotide *de novo* biosynthesis, and acetylene, galactose, and stachyose degradation (*P* < 0.05). The C group had significantly higher relative abundances of genes encoding pathways related to tRNA charging and purine ribonucleoside degradation (*P* < 0.05). The A group had significantly higher relative abundances of genes encoding pathways involved in inosine-5′-phosphate biosynthesis II, 5-aminoimidazole ribonucleotide biosynthesis II, and superpathway of purine nucleotide *de novo* biosynthesis I (*P* < 0.05).

**Fig 6:**
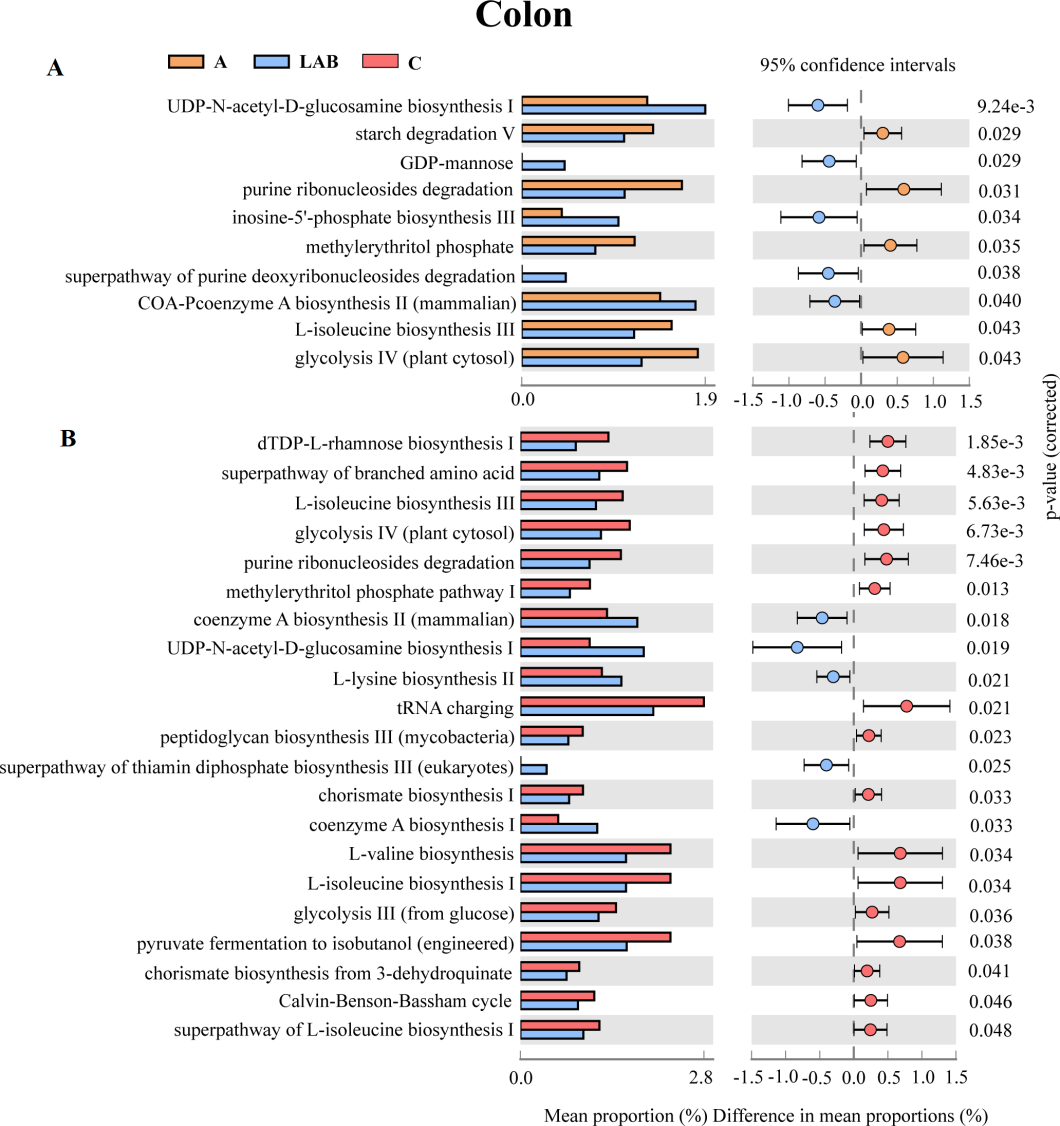
Differentially abundant metabolic pathways encoded in the colonic microbial functional metagenomes. Groups: C, negative control not provided antibiotics or probiotics; A, antibiotics (chlortetracycline) given orally; LAB, probiotic lactic acid bacteria (*Lactiplantibacillus plantarum* P-8) given orally. Pairwise comparisons between the (**A**) A and LAB groups and (**B**) LAB and C groups. No significant differentially abundant pathway was identified between the colonic microbial functional metagenomes of the A and C groups.

For the colonic microbiota (Fig. 6), no significant differential pathway was identified between the C and A groups, and their common pathways were glycolysis and L-isoleucine biosynthesis. By contrast, there were 10 differentially abundant metabolic pathways between the A and LAB groups (Fig. 6A), and 21 between the C and LAB groups (Fig. 6B, all corrected *P* < 0.05). Compared to the C and A groups, the colonic microbiota of the LAB group had significantly higher relative abundances of genes encoding pathways related to GDP-mannose biosynthesis, coenzyme A biosynthesis I, UDP-N-acetyl-D- glucosamine biosynthesis I, L-lysine biosynthesis II, and inosine-5′-phosphate biosynthesis III.

These modulations in the functional metagenomes of the ileal and colonic microbiota could be part of the mechanism that enhanced nutrient absorption and assimilation, and increased energy acquisition, thereby improving the growth performance and immunity in piglets supplemented with probiotics.

### Comparison of gut ARG profiles among the treatment groups

**Fig 7:**
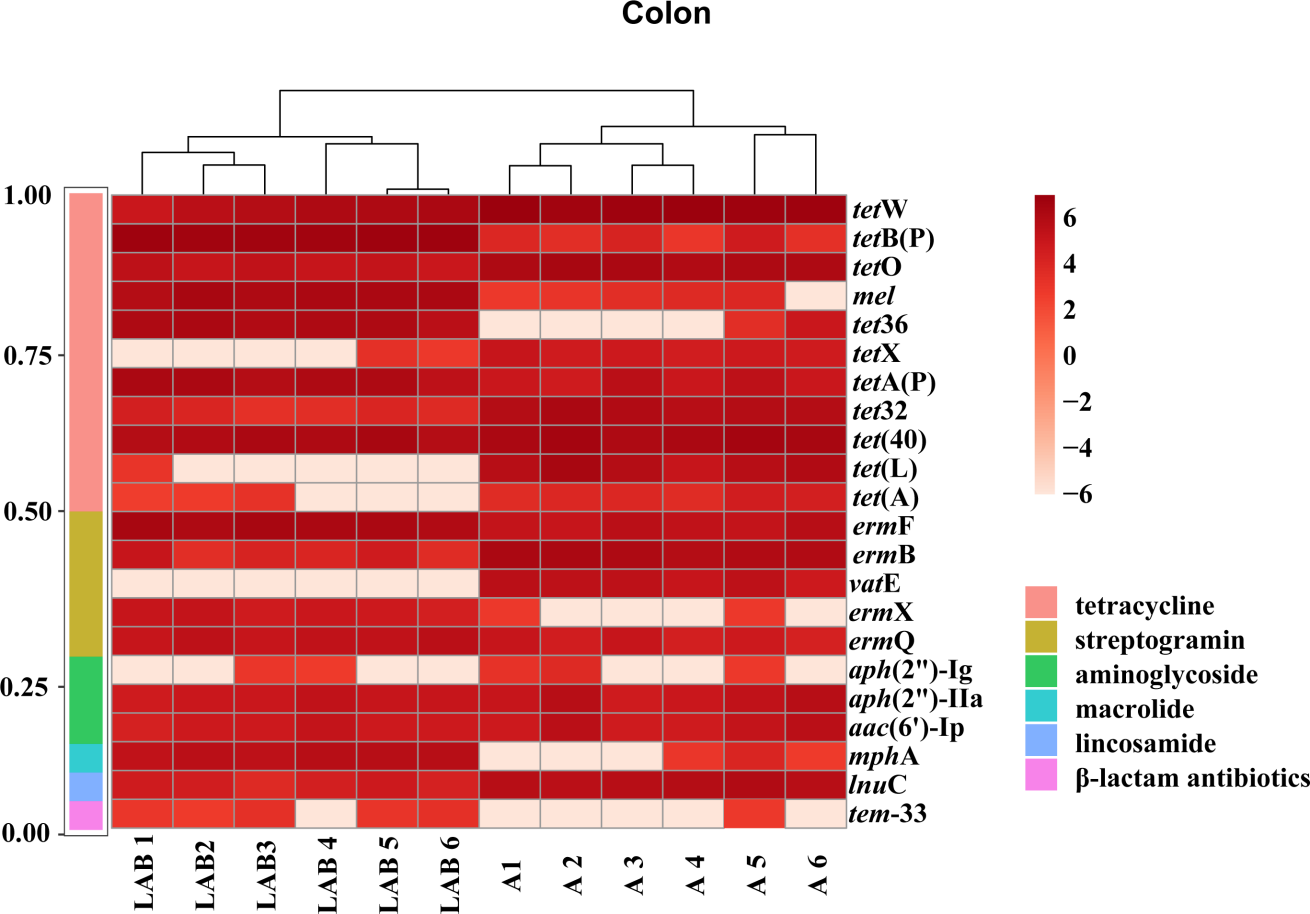
Heatmap showing fold changes in different antibiotic resistance genes (ARGs) detected in the colonic microbial metagenomes of probiotic and antibiotic treatment groups. Each row represents an ARG, and each column represents the colonic ARG profile of one piglet. Each piglet is represented by a different number, and the prefixes “LAB” and “A” represent piglets that received probiotic lactic acid bacteria (*Lactiplantibacillus plantarum* P-8) or an antibiotic (chlortetracycline), respectively. The right-axis color scale represents fold changes (ranging from 6 to −6), and the left-axis color scale (normalized to the sum of 1) represents the proportion of ARGs conferring specific drug resistance (represented by different colors).

The diversity and abundance of ARGs in the ileum did not significantly differ across the three treatment groups. Twenty-two significantly differential ARGs were identified between the colonic microbiota of the LAB and A groups (*P* < 0.05, Fig. 7). Most of these ARGs encoded resistance to antibiotics commonly used in large-scale pig farming, such as tetracyclines, sulfonamides, macrolides, and quinolones. There were some intragroup variation in these differentially abundant ARGs. Moreover, the degree of variation across the treatment groups differed among genes. For example, *tet*X, *tet*(A), *tet*(L), and *vat*E expressions were significantly greater in the colonic microbiome of the A group than the LAB group (*P* < 0.05) whereas *tet*36, *erm*X, *mph*A, and *TEM*-33 showed the opposite trend (*P* < 0.05). These results show that probiotic supplementation modulates the colonic ARG profile in weaned piglets, altering genes related to resistance to antibiotics commonly used in pig farming.

## DISCUSSION

LABs are potential alternatives to antibiotics, as extensive use of antibiotics has driven emerging global concerns of drug resistance. Weaning is the most stressful event in the pig life cycle, impacting piglet health, diminishing growth performance, and causing problems such as gut dysbiosis, oxidative stress, and immune dysfunction. Given that the gut microbiota is closely associated with overall host health and that probiotics can promote host health and maintain gut homeostasis, supplementation with probiotics offers a potential strategy for ensuring a less stressful weaning transition. This study investigated the beneficial effects of the supplementation of weaned piglets with the probiotic strain *L. plantarum* P-8 on their growth performance and immune and antioxidative responses. The ileal and colonic microbiota of the piglets were comparatively analyzed after the intervention.

The provision of *L. plantarum* P-8 to weaned piglets offered several benefits. First, after the 28-day intervention, probiotic-fed piglets showed improved growth performance, as evidenced by improvements in BW, ADG, ADFI, FCR, and *V*:*C* ratio compared to the C group. Meanwhile, BW and ADG did not significantly differ between the A and C groups. Our results are consistent with a previous study that found that *L. plantarum* supplementation improved the growth performance of piglets (25). Probiotics may promote health by inhibiting opportunistic pathogen growth and augmenting nutrient absorption by increasing the *V*:*C* ratio (26).

Second, probiotic-fed piglets exhibited significant improvements in some hepatic antioxidant markers, including higher SOD and GSH-Px activities. Antioxidant enzymes, including T-AOC, SOD, GSH-Px, and CAT, are important components of the body’s antioxidant systems and play a vital role in self-defense (27). Li et al. (28) reported that administering *Lactobacillus delbrueckii* increased the serum activities of T-AOC and CAT as well as hepatic activities of GSH-Px and CAT in piglets, although no significant effects were observed in SOD activity in either the serum or liver. Another study observed higher SOD, CAT, and GSH-Px activities in piglets supplemented with compound *Lactobacillus* sp. than the C group, while no differences were observed in SOD or CAT activities between piglets in the A and C groups (29). Although we found that *L. plantarum* P-8 supplementation strengthened liver but not serum SOD and GSH-Px activities, such findings still suggest that administering *L. plantarum* P-8 could enhance the antioxidant defense system in weaned piglets. It would be valuable to perform further animal trials to investigate whether such effects could be strengthened by a larger probiotic dose.

Piglets can experience significant biological stresses, including physiological, environmental, and social challenges, in response to separation from their mother and familiar littermates, transfer among groups, new surroundings, and dietary changes during the weaning period, resulting in nutritional and health problems accompanied by gut dysbiosis (30, 31). A growing body of research indicates that administering beneficial microbes may help rebalance the gut microbiota, promote growth, and reduce the risk of infectious and post-weaning diarrhea (32–34). Thus, in the present study, we analyzed the impacts of administering a probiotic to weaning piglets on their ileal and colonic microbiota, as well as their functional gut metagenome.

Our results revealed differential treatment-induced responses in the microbial diversities of the ileal and colonic microbiota. At the end of the trial, no significant differences were observed in either the Shannon or Simpson’s diversity index of the ileal microbiota across the C, A, and LAB groups. By contrast, the alpha diversity of the colonic microbiota was significantly lower in the LAB group than in the C and A groups; despite this decrease, the median Shannon and Simpson’s diversity indices were higher than those in the ileal microbiota. Normally, the porcine colon hosts a more diverse microbiota than the ileum due to intrinsic differences in their physical environments and physiological roles (35). Thus, even if homeostasis of the gut microbiota was temporarily disturbed by external stimuli like probiotic or antibiotic treatment, the alpha diversity of the colonic microbiota remained greater than that of the ileal microbiota.

Apart from the different physical environments and biological functions of these two compartments of the gastrointestinal tract, the interactions between the treatment and the intrinsic microbial variety and microenvironment might also contribute to the observed differential responses. The antibiotic-induced decrease in the alpha diversity of the ileal microbiota was much greater than that of the colonic microbiota, although it was non-significant in both cases. The slighter decrease in the microbial diversity of the colonic microbiota could reflect a stronger resilience contributed by its originally higher microbiota diversity and complexity compared to the ileal microbiota. Interestingly, probiotic supplementation significantly decreased the alpha diversity of the colonic microbiota compared to the other two groups. This contradicts a previous study that reported an increased Simpson’s diversity index in piglet microbiota after dietary supplementation with *L. plantarum* PFM105 and *Enterococcus faecium*. It is generally thought that a higher gut microbiota diversity is desirable, but this perspective has been questioned by Shade (36), noting that a high diversity is not necessarily “better” or “healthier” but rather “a starting point for further inquiry into ecological mechanisms rather than an ‘answer’ to community outcomes.” Another possible explanation for the current observation of divergent responses in these two intestinal compartments toward the probiotic intervention could be related to a higher adaptability, survival, and colonization of *L. plantarum* P-8 in the colon, enabling it to eventually become a dominant microbe, compared to the ileum. Beta diversity analysis revealed greater uniformity across the ileal and colonic microbial communities after dietary probiotic or antibiotic supplementation compared to piglets receiving only the basal diet, suggesting that *L. plantarum* P-8 or antibiotic provision could promote intestinal maturation and thus help establish stable microbial communities during the post-weaning phase.

Next, intervention-induced taxonomic differences in the gut microbiota were analyzed to determine the effects of dietary intervention on the evolution of the post-weaning intestinal microbiota. At the phylum level, both the ileal and colonic microbiota were made up of mainly Firmicutes (ileum, 95.16%; colon, 93.10%), consistent with data reported by Pajarillo et al. (37) that the phylum Firmicutes dominated in Landrace pigs; however, it was less abundant in Duroc or Yorkshire pigs, confirming a link between the gut microbiota composition and host factors like genetics and diet. The phylum- and genus-level taxonomic annotations of the ileal and colonic microbiota were in good agreement. The major genus identified in both microbiota was *Lactobacillus*. Interestingly, the probiotic and antibiotic treatments resulted in similar changes in phylum- and genus-level ileal and colonic microbiota, characterized by significant, or at least apparent, increases in *Lactobacillus* (phylum Firmicutes). The increase was greatest in the LAB group. We speculated that the increase in *Lactobacillus* in the LAB group was a direct result of *L. plantarum* P-8 supplementation, which supplied the piglets in the LAB group a large amount of live probiotics to their gastrointestinal tract. Our speculation was supported by the results of LEfSe that *L. plantarum* was greatly enriched in both the ileum and the colon in the LAB group. Likewise, a previous study found that supplementing compound strains of *Lactobacillus salivarius* and *L. reuteri* increased the proportions of *Lactobacillus* in the ileum and cecum of piglets (26).

In the present study, chlortetracycline was administered to piglets as a positive control; tetracyclines and sulfonamides are the most common veterinary antibiotics used in concentrated swine feeding operations in China (38). We identified 22 significantly differential ARGs between the colonic microbiota of the LAB and A groups. The antibiotic treatment caused a detectable increase in the abundance and variety of tetracycline-resistance genes, suggesting that modulation of the gut microbiota was driven by chlortetracycline administration. A previous study on broiler chickens demonstrated that administering *L. plantarum* P-8 modulated the intestinal ARG pool by changing the population structure of the gut microbiota (39). Thus, the differential ARG profiles of the LAB and A groups were likely directed dually by the antibiotic selection pressure for microbes that carry ARG components and the *L. plantarum* P-8-driven gut microbiota modulatory effect.

As anticipated, the apparent increases in the proportions of intestinal *Lactobacillus* were accompanied by decreases in co-occurring taxa including *Actinobacillus* (phylum Proteobacteria) in both the ileal and colonic microbiota; these included Peptostreptococcaceae (phylum Firmicutes) and *Enterocytozoon* (phylum Microsporidia) in the ileal microbiota, and *Phascolarctobacterium*, *Subdoligranulum*, and *Faecalibacterium* (all Firmicutes) in the colonic microbiota. Most of these taxa are gastrointestinal commensals in pigs (40), which naturally fluctuate with changes in developmental stage, growth environment, and diet. Interestingly, *Actinobacillus* decreased in the LAB and A groups, particularly in the ileal microbiota. The results of LEfSe also revealed that *Actinobacillus minor* abundance was diminished in the LAB and A groups compared to the C group. Piglets with high proportions of *Actinobacillus* are more prone to pneumonia and pleuritis (41). Another genus that was beneficially suppressed in the LAB and A groups was *Enterocytozoon. Enterocytozoon* species are obligate intracellular parasitic fungi that cause life-threatening infections in immunocompromised patients. Members of this genus are highly prevalent in pigs (up to 89.5% in a cohort of healthy pigs aged 2–3 months randomly sampled in three regions in Heilongjiang Province, China), serving as asymptomatic carriers/reservoirs of these parasites in nature and greatly increasing the risk of transmission of human microsporidiosis (42). Tetracycline derivatives are only partially effective against microsporidia and would require a toxic dose to be effective (43); thus, the suppressive effect on microsporidia was unlikely the result of a direct “cidal” action of the applied antibiotics. We found that *Lactobacillus acidophilus* CH1-derived bacteriocin effectively inhibited intestinal microsporidiosis when applied together with gold nanoparticles (44). Our observations of increased lactobacilli abundances in both the ileal and colonic microbiota of the A and LAB groups, in particular significant enrichment of *L. reuteri* and *L. plantarum*, respectively (as revealed by LEfSe), suggest that lactobacilli might play a role in reducing microsporidia levels in the piglet gut. *L. reuteri* is an important intestinal microbe and a feed-grade probiotic (45) which has been shown to have multiple beneficial effects after colonizing and residing in the intestinal mucous membrane, such as inhibiting pathogens, restoring gut microbiota composition, enhancing host immunity, digestive function, and intestinal barrier function (46). The enrichment of *L. reuteri* in the A group also suggested that the applied antibiotics inhibited the growth of harmful bacteria but not *L. reuteri*, allowing *L. reuteri* to exert probiotic effects in the host.

Finally, *C. glycolicum* and *V. parvula* were significantly enriched in the ileal microbiota of the C group compared to the other experimental groups. The physiological roles of *C. glycolicum* and *V. parvula* in pigs are yet to be determined, but they are opportunistic pathogens in humans. *C. glycolicum* has been isolated from human wounds, peritoneal fluid, and feces, and potentially acts as a co-infective causative agent in immunocompetent individuals (47). *V. parvula* is part of the normal commensal communities of the mouth, gastrointestinal tract, and vagina in humans but has been implicated as a pathogen in infections of the sinuses, lungs, heart, bone, and central nervous system (48). Therefore, both probiotic and antibiotic supplementation seem to beneficially reduce undesirable gut microbes and improve the intestinal microbiota structure in piglets.

In conclusion, dietary supplementation with *L. plantarum* P-8 may ameliorate stress and improve antioxidant capacity and growth performance in weaned piglets, accompanied by beneficial changes to the gut microbiota. Antibiotics are commonly used to alleviate weaning stress in pig husbandry, and our study suggests that *L. plantarum* P-8 can be used as a substitute for antibiotics to avoid the global concern of drug resistance.

## MATERIALS AND METHODS

### Animals, diets, and sampling

A total of 90 normal weaned piglets (45 males and 45 females) from 11 litters (Landrace × large white, 25 days of age, 7.75 ± 0.08 kg) were allocated randomly to three groups (five replicates per group, six piglets per replicate) for the 28-day trial. As a control, the C group included pigs fed a corn–soybean meal basal diet without any antibiotics or probiotics [composition and nutrient levels shown in Table S1, according to references (49)]. The A group was fed the basal diet plus the antibiotic chlortetracycline (75-mg/kg BW). The LAB group was fed the basal diet plus lyophilized *L. plantarum* P-8 at a final concentration of 1 × 10^8^ CFU/g. All pigs in this study were selected from one delivery room and were similar in their genetic background and husbandry practices; the details of each piglet are shown in Table S2. The three groups of piglets were housed in three large housing rooms within the same housing building at the Ministry of Agriculture Feed Industry Centre in Hebei Province, China. Each room contained multiple pig pens, and each replicate of the same group (five replicates per group, six piglets per replicate) was housed in a single pig pen. The ambient conditions of the housing rooms were controlled: room temperature maintained between 22°C and 26°C; and relative humidity maintained between 60% and 70%.

All piglets were weighed individually at days 0, 14, and 28. The piglets had *ad libitum* access to feed, but the amount of feed consumed by the piglets in each pen was monitored daily. The ADG, ADFI, and FCR were each calculated for three periods: days 1–14, 15–28, and 1–28.

At day 28, 10-mL blood was sampled from the piglet weighing closest to the median BW in each replicate, and the piglet was sacrificed; samples of liver and ileal mucosa tissues, along with colon and ileum contents, were taken and transferred immediately to liquid nitrogen. Then, all samples were sent to the laboratory, where they were stored at −80°C until further analysis.

### Determination of physiological parameters and immune markers

#### Detection of antioxidant indices in serum and liver

Serum samples were separated by centrifugation at 3,000 rpm for 15 min at 4°C. For protein extraction, 0.5-g liver and ileal mucosa tissues were homogenized in 450-µL 0.9% NaCl. The T-AOC, CAT, SOD, GSH-Px, and MDA levels in the serum, liver tissue, and ileal mucosal samples were determined using off-the-shelf kits (Nanjing Jiancheng Bioengineering Institute, Nanjing, China). The serum concentrations of IgG, IgA, IgM, TNF-α, IL-2, IL-4, IL-6, IL-10, IL-1β, and IFN-γ were measured using sandwich enzyme-linked immunosorbent assay kits (Nanjing Jiancheng Bioengineering Institute) according to the manufacturer’s protocols. Their concentrations were then calculated from the standard curves. All procedures were performed in triplicate.

#### Histological analyses

The ileal mucosa tissues collected from the sacrificed piglets at day 28 were dehydrated, embedded in paraffin, and sectioned for intestinal morphological assessment, as previously described (50). The villus height and crypt depth in the ileal segments were measured with Image-Pro software (Media Cybernetics, Rockville, MD, USA).

### Whole-genome metagenomics sequencing of ileal and colonic microbiota

#### Genomic DNA extraction and sequencing

The QIAamp DNA Stool Mini Kit (Qiagen, Hilden, Germany) was used to extract DNA from the ileum and colon contents. The quality of the extracted DNA was checked by agarose gel electrophoresis and spectrophotometric analysis (optical density ratio: 260 nm/280 nm). All DNA samples were stored at −20°C prior to further experiments. Metagenomic DNA libraries were constructed with 2-µg genomic DNA according to the Illumina TruSeq DNA Sample Prep Guide (v.2), and the quality of DNA libraries was assessed using an Agilent 2100 Bioanalyzer with a DNA LabChip 1000 kit (Agilent, Santa Clara, CA, USA). Then, sequencing was carried out using the HiSeq X Ten instrument (Illumina, San Diego, CA, USA). The raw paired-end reads were quality controlled according to the criteria reported previously (24). The high-quality reads were retained and used for subsequent analyses.

#### Bioinformatics analysis

The extracted data were analyzed using the HUMAnN2 software tool suite (51). MetaPhlan 2 (v.2.0) was used to calculate the taxonomic distribution of the ileal and colonic microbiota. The KEGG database was used for metagenomic pathway analysis and identification of differentially abundant metagenomic pathways among the groups. Kraken2 (52) was applied for annotation of metagenomic species, while the functional metagenome and corresponding metabolic pathways were annotated using the HUMAnN2 pipeline based on the UniRef90 database (https://www.uniprot.org/help/uniref). The metagenome sequence was compared to the Comprehensive Antimicrobial Research Database, and the annotation information on 22 resistance genes was obtained. Seven antibiotics were selected for subsequent analysis according to the abundance and the types of antibiotics commonly used in the pig industry.

PCoA was performed using OmicStudio (https://www.omicstudio.cn). Microbial abundance was compared at the phylum, genus, species, and pathway levels, within and between groups, using the Wilcoxon test in R software. We used STAMP software (v.2.1.3) and LEfSe (http://huttenhower.sph.harvard.edu/galaxy/) to screen and visualize differentially abundant taxa and metabolic pathways among the groups (53). Data were plotted using R software (v.4.0.4), Origin 2021 software (OriginLab Corporation, MA, USA), and GraphPad Prism (v.8.0.2; GraphPad Software Inc., La Jolla, CA, USA).L

### Statistical analyses

The ADG, ADFI, FCR, and relative abundance data were analyzed using linear mixed-effects models in R (v.4.0.4). *P* values of < 0.05 (with Bonferroni adjustment) were considered significant. Results are presented as the means ± standard deviation. Alpha diversity parameters were compared between time points using the Wilcoxon test. Two-way analysis of variance was applied to investigate longitudinal differences of the Bray–Curtis and Jaccard distances between littermates and litters. The statistical significance of ANOSIM was determined based on 999 permutations between dietary categories. The *R* value was calculated using the following formula: *R* = difference of mean rank (all distances between groups − all distances within groups) / [N(N − 1) / 4]. *R* values range between 0 and 1; larger values reflect greater dissimilarity between groups. The *P* value represents the significance level.

## Data Availability

Data sets generated by whole-genome metagenomics sequencing were deposited in the National Center for Biotechnology Information Short Read Archive database under the accession number PRJNA781781.

## SUPPLEMENTAL MATERIAL

Supplemental material spectrum.02345-22-s0001.pdf Table S1 and Table S2.

ASM does not own the copyrights to Supplemental Material that may be linked to, or accessed through, an article. The authors have granted ASM a non-exclusive, world-wide license to publish the Supplemental Material files. Please contact the corresponding author directly for reuse.
