# Supplementary material for: Dietary supplementation with Lactiplantibacillus plantarum P-8 improves the growth performance and gut microbiota of weaned piglets
Source: Microbiol Spectr. 2024 Jan 3;12(2):e02345-22. doi: 10.1128/spectrum.02345-22 (PMC10845957; doi:10.1128/spectrum.02345-22)
Supplement: Supplemental material — Table S1 and Table S2. [file spectrum.02345-22-s0001.pdf]

Table S1 Composition and nutrient levels of the corn-soybean meal basal diet

Items (%)	Basal diet
Composition	
Corn	59.59
Soybean meal	15.00
Full fat soybean	10.00
Fish meal	4.00
Whey powder	5.00
Soybean oil	3.00
Dicalcium phosphate	1.00
Limestone	0.80
Sodium chloride	0.25
L-Lysine·HCl	0.47
DL-Methionine	0.16
L-Threonine	0.20
L-Tryptophan	0.03
Premix	0.50
Nutrient levels	
Analyzed values	
Crude protein	18.34
Calcium	0.72
Total phosphorus	0.57
Calculated values	
Digestible energy (kcal/kg)	3497
SID Lysine	1.24
SID Methionine	0.36
SID Threonine	0.74
SID Tryptophan	0.22

Note:[1] Premix provided the following per kg of feed: vitamin A, 12,000 IU; vitamin D_3, 2,500 IU; vitamin E, 30 IU; vitamin K_3, 30 mg; vitamin B_{12}, 12 μg; riboflavin, 12 mg; pantothenic acid, 15 mg; niacin, 40 mg; choline chloride, 400 mg; folic acid, 0.7 mg; vitamin B_1, 1.5 mg; vitamin B_6, 3 mg; biotin, 0.1 mg; Mn, 40 mg; Fe, 90 mg; Zn, 100 mg; Cu, 8.8 mg; I, 0.3 mg; Se, 0.3 mg.

[2] Standardized ileal digestible, SID.

Table S2 Detailed information of weaning piglets

LAB group				Control group				Antibiotics group			
Pen	Sample	Sexuality	Pig	Pen	Sampl	Sexuality	Pig ID	Pen	Sample	Sexuality	Pig ID
1	L1-1	female	[a]B6	2	C1-1	female	A4	3	A1-1	male	A11
1	L1-2	female	F6	2	C1-2	female	A5	3	A1-2	male	A15
1	L1-3	male	G5	2	C1-3	male	A8	3	A1-3	female	A2
1	L1-4	female	H9	2	C1-4	male	G7	3	A1-4	female	F9
1	L1-5	male	I5	2	C1-5	male	H5	3	A1-5	female	G3
1	L1-6	male	L11	2	C1-6	female	J1	3	A1-6	male	I4
4	L2-1	female	A1	5	C2-1	female	B1	6	A2-1	male	A14
4	L2-2	male	A13	5	C2-2	male	B13	6	A2-2	female	A3
4	L2-3	male	F5	5	C2-3	female	D8	6	A2-3	male	C1
4	L2-4	female	G2	5	C2-4	male	F2	6	A2-4	female	C4
4	L2-5	male	G6	5	C2-5	male	I9	6	A2-5	female	F3
4	L2-6	female	I8	5	C2-6	female	J2	6	A2-6	male	G9
7	L3-1	female	B5	8	C3-1	female	A6	9	A3-1	male	C2
7	L3-2	female	B7	8	C3-2	female	F11	9	A3-2	female	G11
7	L3-3	male	F4	8	C3-3	female	G1	9	A3-3	female	H10
7	L3-4	male	H1	8	C3-4	male	G10	9	A3-4	male	K10
7	L3-5	male	I6	8	C3-5	male	H2	9	A3-5	female	K3
7	L3-6	female	K11	8	C3-6	male	K9	9	A3-6	male	L10
10	L4-1	female	B4	11	C4-1	male	F10	12	A4-1	male	B11
10	L4-2	female	C3	11	C4-2	female	J3	12	A4-2	female	D3
10	L4-3	male	D7	11	C4-3	male	K5	12	A4-3	male	H3
10	L4-4	male	F8	11	C4-4	female	L12	12	A4-4	female	H8
10	L4-5	male	I7	11	C4-5	female	L8	12	A4-5	male	I3
10	L4-6	female	L2	11	C4-6	male	L9	12	A4-6	female	L7
13	L5-1	male	B9	14	C5-1	male	B10	15	A5-1	female	B3
13	L5-2	female	D5	14	C5-2	male	B12	15	A5-2	female	D1
13	L5-3	male	K2	14	C5-3	female	B2	15	A5-3	male	D2
13	L5-4	female	K4	14	C5-4	male	H6	15	A5-4	female	D6
13	L5-5	male	L3	14	C5-5	female	L5	15	A5-5	male	K6
13	L5-6	female	L4	14	C5-6	female	L6	15	A5-6	male	L1

Note:[1] the first capital letter represent the sows number, the second number represent different piglets of the sows.
